# *SDHx* and Non-Chromaffin Tumors: A Mediastinal Germ Cell Tumor Occurring in a Young Man with Germline *SDHB* Mutation

**DOI:** 10.3390/medicina56110561

**Published:** 2020-10-25

**Authors:** Giuseppina De Filpo, Antonio Cilotti, Luigi Rolli, Ugo Pastorino, Angelica Sonzogni, Silvia Pradella, Giulia Cantini, Tonino Ercolino, Gabriella Nesi, Massimo Mannelli, Mario Maggi, Letizia Canu

**Affiliations:** 1 Department of Experimental and Clinical Biomedical Sciences “Mario Serio”, University of Florence, 50139 Florence, Italy; giulia.cantini@unifi.it (G.C.); massimo.mannelli@unifi.it (M.M.); mario.maggi@unifi.it (M.M.); letizia.canu@unifi.it (L.C.); 2 Endocrinology Unit, Careggi University Hospital, 50139 Florence, Italy; antonio.cilotti@unifi.it (A.C.); tonino.ercolino@unifi.it (T.E.); 3 Division of Thoracic Surgery, IRCCS Foundation, Istituto Nazionale dei Tumori, 20133 Milan, Italy; luigi.rolli@istitutotumori.mi.it (L.R.); ugo.pastorino@istitutotumori.mi.it (U.P.); 4 Department of Pathology and Laboratory Medicine, IRCCS Foundation, Istituto Nazionale dei Tumori, 20133 Milan, Italy; angelica.sonzogni@istitutotumori.mi.it; 5 Department of Radiology, Careggi University Hospital, 50139 Florence, Italy; pradella3@yahoo.it; 6 Department of Health Sciences, Division of Pathological Anatomy, University of Florence, 50139 Florence, Italy; gabriella.nesi@unifi.it

**Keywords:** SDHx, immunohistochemistry, genetic analysis, GCT

## Abstract

*Background:* Mutations in genes encoding one of the subunits of succinate dehydrogenase (SDH) are involved in pheochromocytoma (PHEO) and paraganglioma (PGL) development. Over the last few years, such mutations have also been associated with non-chromaffin tumors. However, immunohistochemistry (IHC) on the tumor tissue and a study on the loss of heterozygosity (LOH) aimed at demonstrating the pathogenic role of *SDHx* genes have only been employed in a few cases. *Case report:* We describe the case of a 19-year-old Caucasian man with a germline *SDHB* mutation, who presented with acne *vulgaris* resistant to medical treatment. His follow-up for chromaffin tumors was negative, while hormonal tests revealed suppressed gonadotropins with testosterone in the upper range of normality and elevated β-human chorionic gonadotropin (β-hCG). At the whole-body enhanced CT scan, a mediastinal lesion suggestive of a germ cell tumor (GCT) was detected. ^18^FDG-PET (fluorodeoxyglucose-positron emission tomography) imaging showed low glucose metabolism at the mediastinal site. Surgical removal of the mass was uneventful. Pathology confirmed the diagnosis of GCT consisting of cystic teratoma (95%) and seminoma (5%). IHC for SDHB showed normal protein expression, and genetic analysis of the tumor tissue revealed the absence of *SDHB* LOH. Normalization of the hormonal tests and acne attenuation were achieved after surgery. *Conclusion:* We report an incidental association of a germinal *SDHB* mutation and mediastinal GCT in a young Caucasian man. Our paper highlights the importance of IHC and genetic analysis in confirming the etiologic role of *SDHx* genes in nonchromaffin tumors, thus excluding incidental associations.

## 1. Introduction

Pheochromocytomas (PHEOs) and paragangliomas (PGLs) are rare neural crest-derived tumors [1]. PHEOs arise from chromaffin cells of the adrenal medulla and generally release catecholamines. PGLs originate from chromaffin cells of sympathetic ganglia of the thorax, abdomen and pelvis or from parasympathetic ganglia of the head and neck (HNPGL), which generally do not produce catecholamines [2].

Up to 50% of these tumors are caused by germline or somatic mutations occurring in one of the susceptibility genes, including those encoding succinate dehydrogenase (SDH) [3,4,5].

SDH, or mitochondrial complex II, is involved in the Krebs cycle, oxidizing succinate into fumarate, and in the electron transport chain. The enzymatic complex consists of four subunits, i.e., A, B, C and D, encoded by the corresponding genes (SDHA, SDHB, SDHC and SDHD, respectively). The succinate dehydrogenase complex assembly factor 2 (SDHAF2) subunit, encoded by the SDHAF2 gene and responsible for the flavination of subunit A, is also required for the complex enzymatic activity. Mutations of any of these five genes cause inactivation of the SDH complex (*SDHx*) and are involved in the development of familial paragangliomatosis 1–5 [6,7,8].

Interestingly, SDH deficiency has also been associated with non-chromaffin tumors, most frequently, gastrointestinal stromal tumors (GISTs) [9,10,11], renal cell carcinomas (RCCs) [12,13,14] and pituitary adenomas (PAs) [15,16,17]. Among the reported neoplasms [17,18,19], only one case of germ cell tumor (GCT) has been described [20].

Immunohistochemistry (IHC) for SDHB is a reliable and inexpensive method to identify impaired SDH expression in tumor tissues, showing high sensitivity and specificity [21,22]. In particular, a germline mutation of *SDHx* destabilizes the complex, resulting in the loss of SDHB expression at IHC in *SDHB*, *SDHC* and *SDHD*-mutated tumors. On the contrary, the loss of SDHA expression is only found in *SDHA*-mutated tumors [21], suggesting that negative immunohistochemical staining for SDHB can be employed as a surrogate marker for SDH mutations.

Genetic tests on patient blood samples and tumor tissues can be used to confirm the immunohistochemical results of the *SDHx* germinal mutations, as well as to screen for the loss of heterozygosity (LOH).

## 2. Case Presentation

A 19-year-old Caucasian man was referred to our unit for acne *vulgaris* resistant to medical therapy in July 2019. There was a family history of paragangliomatosis 4, due to a *SDHB* mutation (c.470T > G, p.Leu157Trp, exon 5) from the mother. No other familial diseases were recorded.

On clinical examination, the patient showed cystic acne on his face and back, a slightly reduced testicular volume (left testis 12 mL and right testis 10 mL) and no gynecomastia. No anosmia, headache or visual field defects were reported. No medication had been prescribed. In view of the patient’s family history, a genetic analysis was performed to assess the presence of the *SDHB* known mutation.

**Figure 1:**
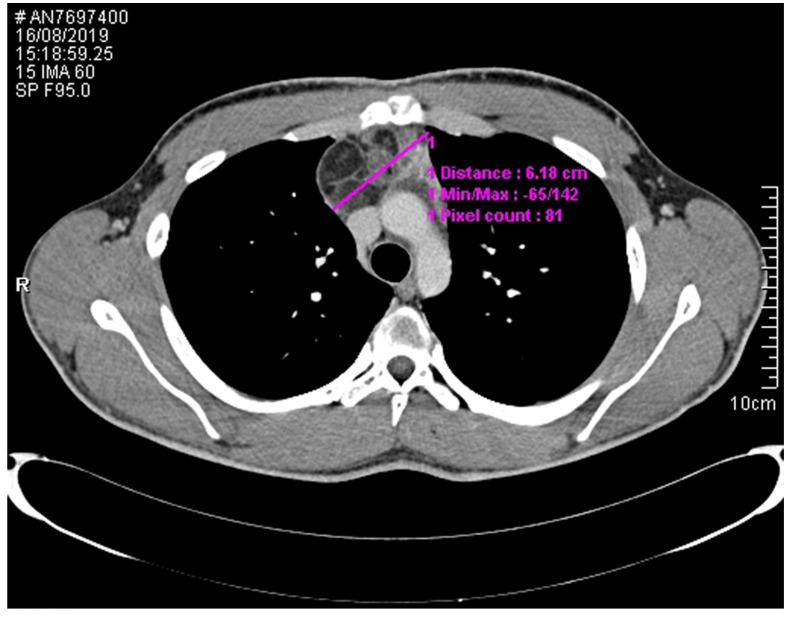
CT scan demonstrating a germinal cell tumor (GCT) in the anterior mediastinum.

Both urinary metanephrines and chromogranin A were negative. Hormonal tests, carried out to establish the causes of acne, showed suppressed gonadotropins (follicle-stimulating hormone (FSH) <0.3 mUI/mL—nv 1.5–12.4 and luteinizing hormone (LH) <0.2 mUI/mL—nv 1.7–8.6), testosterone levels in the upper range of normality (32 nmol/L—nv 4.1–32.9) and increased levels of β-human chorionic gonadotropin (β-hCG) (35.4 U/L—nv 0.1–2). The remaining pituitary functions were normal; lactic dehydrogenase (LDH) and α-fetoprotein (αFP) were not elevated (121 UI/L—nv 135–225 and 1.7 U/mL—nv 0–5.8, respectively). No adrenal or testicular lesions were detected at ultrasonography. A whole-body enhanced CT scan demonstrated a 6 × 5.8 × 3.6-cm mass in the anterior mediastinum, consistent with GCT (Figure 1). ^18^FDG-PET (fluorodeoxyglucose-positron emission tomography) imaging detected a low glucose uptake in the mediastinum.

In September 2019, the patient underwent surgery without complications. Histology confirmed the presence of mixed GCT consisting of teratoma (95%) and seminoma (5%). After surgery, acne improved while β-hCG, testosterone, LH, and FSH returned to normal levels (Table 1). The patient is on follow-up for germ cell and chromaffin tumors.

## 3. Materials and Methods

### 3.1. Molecular Analysis

After the patient gave his written informed consent, genetic testing was carried out in agreement with our ethical committee guidelines. DNA was extracted from peripheral blood by the QIAsynfony CDN kit (Qiagen S.R.L, Milan, Italy) and screened for the mother’s mutation, c.470T > G, pLeu157Trp, using coding regions and exon-intron boundaries on exon 5 of the *SDHB* gene for PCR amplification. An amount of 50 ng/μL of DNA was processed. After PCR product purification, treated with the PCR purification kit (Qiagen S.R.L., Milan, Italy) and sequenced by standard direct sequencing with the BigDye version 3.1 kit (Applied Biosystems, Foster City, CA, USA), sequencing reactions were evaluated by a model ABI PRISM 310 genetic analyzer (Applied Biosystems, Foster City, CA, USA).

### 3.2. Loss of Heterozygosity (LOH) Analysis

LOH was performed on DNA extracted from freshly snap-frozen tumor samples collected by the pathologist. Subsequently, 50 ng/μL of DNA was processed as described in the Molecular Analysis section.

### 3.3. Immunohistochemistry (IHC)

Formalin-fixed, paraffin-embedded tissue sections of 3-μm thickness were deparaffinized in Bio-Clear (Bio-Optica Milano S.p.A., Milan, Italy) and hydrated with grade ethanol concentrations until distilled water. Briefly, slides were subjected to heat-induced epitope retrieval in ethylenediaminetetra-acetic acid (EDTA) for 30 min. After washing with peroxidase block (DAKO, Carpinteria, CA, USA) for 10 min to quench endogenous peroxidase activity, sections were incubated with mouse monoclonal anti-SDHB (clone 21A11AE; Abcam, Cambridge, UK; 1:500). Immunohistochemical analysis was carried out using DAKO EnVision FLEX (DAKO, Carpinteria, CA, USA) and 3,3′-diaminobenzidine as the chromogen. The sections were lightly counterstained with Mayer’s hematoxylin (Bio-Optica Milano S.p.A., Milan, Italy).

## 4. Results

**Figure 2:**
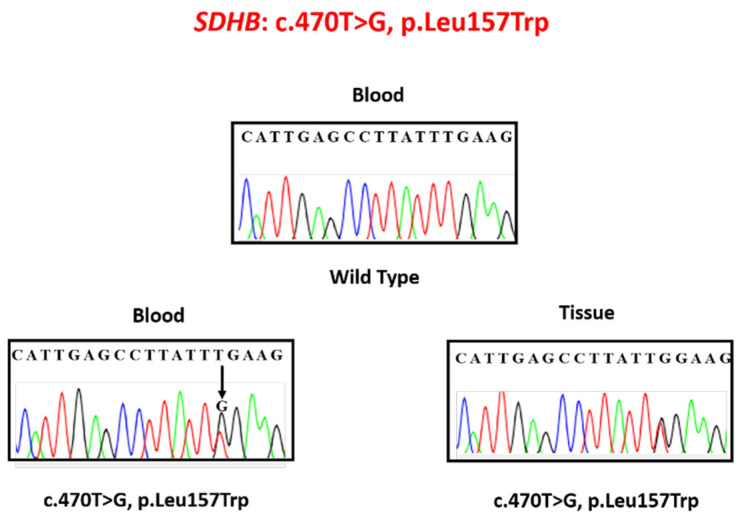
Wild-type (**upper**) and patient (**lower**) electropherograms showing the germline mutation p.Leu175Trp in exon 5 of the *SDHB* gene (**left**) and the somatic mutation without loss of heterozygosity (LOH) (**right**).

The genetic analysis of the patient’s DNA revealed a germline *SDHB* mutation on exon 5, c.470T > G, pLeu157Trp. This heterozygous mutation, a thymine-to-guanine nucleotide substitution, causes a leucine-to-tryptophan amino acid change in *SDHB* codon 157. There was no evidence of LOH, as shown in Figure 2.

**Figure 3:**
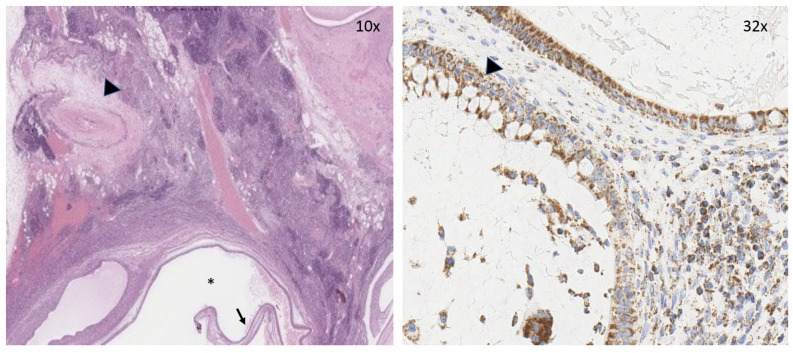
(**Left**) Low-power view shows mature teratoma with cystic structures (*) lined by respiratory-type epithelium (arrow). A Pacinian corpuscle is also evident (arrowhead). (**Right**) Mucous-secreting epithelial cells (arrowhead) retain SDHB positivity at the immunohistochemical analysis (brown precipitate indicates the presence of the target antigen; hematoxylin counterstaining).

IHC for SDHB disclosed diffuse granular staining in the cytoplasm of both neoplastic and non-neoplastic cells (Figure 3).

## 5. Discussion

In recent years, other tumors besides PHEO/PGLs have been linked to *SDHx* mutations—most frequently, GISTs, RCCs and PAs. In particular, *SDH*-deficient GISTs, accounting for 5–7.5% of all GISTs, arise predominantly in the stomach [23] and show similar morphologic and immunohistochemical features as their non-*SDH*-deficient counterparts [10,24]. *SDH*-deficient RCCs constitute 0.05–2% of all RCCs, which are recognized as a distinct type of renal carcinoma, according to the World Health Organization (WHO) 2016 classification and are multifocal and bilateral in 26% of cases [25,26]. *SDHx* associated PAs are usually prolactin (PRL) or somatotropin (GH)-secreting adenomas [27], and no peculiar morphologic characteristics are described. Interestingly, Gill et al. demonstrated SDH deficiency in a case of PA harboring two somatic *SDHA* mutations with no evidence of germline mutation [16]. Other tumors have rarely been reported in SDH mutation carriers; however, IHC or a molecular analysis were hardly ever performed.

To date, *SDHx* deficiency has been demonstrated in only one case of GCT. Miettinen et al. reported an instance of testicular seminoma with negative SDHB staining on immunohistochemical examination, but no molecular or genetic tests were carried out [20]. The current literature does not allow establishing the clinical course of GCTs associated with *SDHx* mutations. However, we could speculate a potentially more aggressive behavior, considering the pseudohypoxia pathway activation leading to increased ROS, angiogenesis and cellular proliferation in *SDH*-deficient tumors [28].

Our patient was an *SDHB* mutation carrier diagnosed with GCT but showing no evidence of chromaffin disease. To address the question of whether the GCT could be due to *SDH* impairment, IHC was performed, demonstrating strong granular cytoplasmic staining for SDHB. A genetic analysis on the tumor tissue gave LOH-negative results. Taking into account these findings, we hypothesized an incidental association between *SDHB* mutation and the occurrence of GCT in our patient. The lack of LOH suggests that genes other than *SDH* may be involved in this disease.

It is common knowledge that mixed GCTs and mediastinal teratomas are more likely to develop in patients with Klinefelter’s syndrome [29,30]. Since clinical features of hypergonadotropic hypogonadism were absent in the present case and hormonal assays normalized after surgery, the patient karyotype was not analyzed.

Based on the limited data available, *SDHx* mutation carriers may also be examined for GCTs, if the clinical history is pertinent. Similarly, in patients with nonchromaffin tumors, it is advisable to investigate for *SDHx* germline mutations if SDHx deficiency is suspected. The immunohistochemical analysis has been proposed as a screening/triaging test [21,22], although SDHA and SDHB IHC should be interpreted with caution because of possible false positive results, particularly in the presence of *SDHD* gene mutations [31,32]. For this reason, a genetic analysis on the blood and/or tumor tissues is essential to confirm the immunohistochemical findings and detect the germline mutations.

## 6. Conclusions

Our paper highlights the importance of IHC and LOH analyses in establishing the pathogenic role of *SDHx* genes in patients with non-chromaffin tumors and SDHx impairment. In particular, in our patient, no pathogenetic role of *SDHB* mutation was demonstrated in GCT development, implying that other susceptibility genes may play a role in this tumor type.

Further investigations are required to elucidate the clinical characteristics of GCTs associated with *SDHx* mutations and to assess any differences in biological behavior from non-*SDHx*-related GCTs.

## Figures and Tables

**Table 1:** Patient hormonal evaluation before and after surgery for mediastinal GCT (germ cell tumor). LH (luteinizing hormone), FSH (follicle-stimulating hormone) and β-hCG (β-human chorionic gonadotropin).

	LH (nv 1.7–8.6 mUI/mL)	FSH (nv 1.5–12.4 mUI/mL)	Testosterone (nv 4.1–32.9 nmol/L)	β-hCG (nv 0.1–2 U/L)
Before surgery	<0.3	<0.3	32	35.4
After surgery	7.4	5.1	22	0.1
